# Amino Compound-Synthesized Gold Nanoparticles for SARS-CoV-2 Antigen Delivery

**DOI:** 10.3390/pharmaceutics17091211

**Published:** 2025-09-17

**Authors:** Layane Souza Rego, Marianna Teixeira Pinho Favaro, Monica Josiane Rodrigues-Jesus, Robert Andreata-Santos, Luiz Mário Ramos Janini, Marcelo Martins Seckler, Luis Carlos de Souza Ferreira, Adriano Rodrigues Azzoni

**Affiliations:** 1 Departamento de Engenharia Química, Escola Politécnica, Universidade de São Paulo, Av. Prof. Luciano Gualberto, Trav. 3, Nº 380, Butantã, São Paulo CEP 05508-900, SP, Brazil; 2 Laboratório de Desenvolvimento de Vacinas, Departamento de Microbiologia, Instituto de Ciências Biomédicas, Universidade de São Paulo, Av. Prof. Lineu Prestes, 2415-Butantã, São Paulo CEP 05508-000, SP, Brazil; 3 Institut de Biotecnologia i de Biomedicina, Universitat Autònoma de Barcelona, 08193 Bellaterra, Spain; 4 Division of Infectious Diseases and International Health, University of Virginia, 345 Crispell Drive, MR-6, Charlottesville, VA 22908, USA; 5 Laboratório de Retrovirologia, Departamento de Microbiologia, Imunologia e Parasitologia, Escola Paulista de Medicina, Universidade Federal de São Paulo (UNIFESP), São Paulo CEP 04023-062, SP, Brazil

**Keywords:** gold nanoparticles, amino compounds, nanomedicine, vaccines, SARS-CoV-2

## Abstract

**Background:** Gold nanoparticles (AuNPs) are a promising platform for vaccine antigen delivery due to their ability to stimulate both innate and adaptive immune responses. These effects depend strongly on physicochemical properties such as size, polydispersity, morphology, and surface charge, which are in turn determined by the synthesis method. While amino acids are often used as capping agents for AuNPs, their direct use as both reducing and stabilizing agents has been rarely investigated. **Objectives:** This study aimed to establish an ultrasound-assisted method for synthesizing AuNPs using amino compounds as both reducing and stabilizing agents, and assess their physicochemical characteristics, antigen-binding capacity, and immunogenicity. **Methods:** AuNPs were synthesized using L-cysteine, L-arginine, and cysteamine as dual reducing/stabilizing agents under ultrasonic conditions. The nanoparticles were combined with a recombinant receptor-binding domain (RBD) of SARS-CoV-2 and evaluated in mice for their ability to induce antibody responses. **Results:** The synthesized AuNPs exhibited hydrodynamic diameters ranging from 6.3 to 12.4 nm and zeta potentials from −40.5 to +36.5 mV, depending on the amino compound used. All formulations elicited robust anti-RBD IgG responses, but virus neutralization activity varied significantly. Notably, AuNP–arginine induced the strongest neutralizing response despite lower adsorption capacity and stability, suggesting that epitope preservation and antigen presentation quality were more decisive than antigen density. **Conclusions:** These findings underscore the importance of nanoparticle design in optimizing antigen presentation and highlight the potential of amino compound-synthesized AuNPs as effective antigen delivery vehicles for future vaccine development.

## 1. Introduction

The emergence of new infectious diseases has raised the need to establish novel effective and versatile vaccine platforms. Among these new platforms, gold nanoparticles (AuNPs) have become an alternative tool for the development of vaccines due to their biocompatibility, efficient surface functionalization, particle size, and morphology [1,2,3]. Indeed, AuNPs have been employed as both delivery system and adjuvant for improvement of induced antibody and T-cell responses, while ensuring little or no cytotoxicity [1,2,3,4,5,6,7]. Recently, a randomized, double-blind phase 1 trial employing an AuNP-based candidate vaccine, using multi-valent synthetic peptide antigen, showed the induction of protective CD8+ T-cell immunity [8]. Besides, a favorable safety profile and cellular responses were found, supporting further development of the vaccine [8].

When designing an AuNP-based platform, size, morphology, and functional groups have an important impact on antigen association, immune response, cellular uptake, biodistribution, and blood clearance [2,9]. As an adjuvant, AuNPs help to promote or improve the expression of pro-inflammatory cytokines through different pathways, depending on their morphology and mode of administration [9,10]. The size and morphology of the nanoparticles usually have an impact on absorption and biodistribution, while surface coating/functionalization can also change circulation and bioavailability [11,12,13]. Thus, the development of reliable AuNP synthesis and conjugation methods are crucial for the enhancement of the effectiveness of AuNP-based vaccines [12,13].

In view of the aforementioned advantages, in this work we developed an ultrasound-assisted method for AuNP synthesis, producing smaller and narrower size distribution nanoparticles under lower temperatures and shorter reaction times [11]. In this method, the AuNPs were reduced and coated using L-cysteine, L-arginine, and also cysteamine, a decarboxylated derivative of the amino acid cysteine. In general, amino acids have been extensively used to cap AuNPs synthesized with traditional reducing agents, such as sodium citrate or sodium borohydride [14,15]. However, not so many studies have been dedicated to understanding the use of amino acids as reducing and capping agents during AuNPs synthesis [15,16,17]. The use of amino acids has several advantages: a single molecule provides both amine and carboxylic acid groups, enabling conjugation with different biomolecules and enhancing nanoparticle biocompatibility. Figat and colleagues (2023) investigated the use of 21 different amino acids for AuNP synthesis [15]. Only four amino acids (L-proline, L-cysteine, L-glutamine, and L-arginine) failed to produce AuNPs, although all except L-proline reduced gold salts. A reaction mechanism was also proposed by the authors for the synthesis of AuNPs using most of the amino acids studied. One important limitation of the method described is the difficulty of controlling the nucleation process during nanoparticle formation. In contrast, in the present work, we demonstrate that some of these amino acids (L-cysteine and L-arginine) are effective reducing and stabilizing agents when combined with an ultrasound-assisted methodology and the inverse addition of reagents. The synergistic effects of acoustic cavitation, which provides localized high temperatures and pressures, and controlled supersaturation achieved by inverse addition, appear to favor a faster and more uniform nucleation process [18]. These reaction conditions likely overcome the limitations previously described by Figat and colleagues (2023), particularly the tendency of L-cysteine to yield sulfur precipitates [15].

Beyond the synthesis aspects, the functionalization of AuNPs with amino compounds is particularly advantageous for vaccine development. The presence of functional groups such as amine, thiol, and carboxyl moieties not only stabilizes the nanoparticles in aqueous media but also facilitates reversible interactions with protein antigens. This molecular environment is critical for preserving the conformational epitopes of antigens, which represent the main targets of neutralizing antibodies. Therefore, the use of amino compound-stabilized AuNPs provides a dual benefit: enabling efficient nanoparticle synthesis and ensuring favorable surface chemistry for antigen adsorption and immune recognition. This rationale guided the present study, in which we explored the capacity of amino compound-synthesized AuNPs to act as delivery vehicles for antigens, particularly a recombinant form of the receptor-binding domain (RBD) of the spike protein of SARS-CoV-2 [19,20]. The RBD sequence is responsible for mediating the binding of the virus to the host cell angiotensin-converting enzyme 2 (ACE2) [19]. RDB is an immunodominant and highly specific target of antibodies that mainly recognize conformational epitopes present on the surface of the protein expressed by virus particles. Thus, proper antigen conformation is crucial for maintaining the ability to elicit antibodies capable of binding and blocking the ability of virus particles to infect susceptible host cells [2,10]. The results reported herein indicated that different AuNP-RBD formulations were capable of affecting the induction of antibodies with virus neutralizing activity compared to mice immunized with the antigen not associated with AuNP. Thus, the present study reinforces the potential of AuNPs as an antigen delivery platform for the design and production of future protein subunit vaccine prototypes.

## 2. Materials and Methods

### 2.1. Synthesis of AuNPs Reduced and Capped with Amino Compounds

A novel method for AuNP synthesis was developed by combining sonochemistry with the Turkevich inverse addition method [21]. Briefly, 19 mL of a solution of the reducing/capping agent with a concentration of 5.20 mM (pH 7.0) was added to a 100 mL Erlenmeyer flask. The solutions were heated to 80 °C and kept in a 100 W ultrasound bath. The reducing/capping agents were either tribasic sodium citrate dihydrate, L-cysteine, cysteamine, or L-arginine. After the desired temperature was established, 1.0 mL of a 10.4 mM solution of tetrachloroauric acid was added. After the reaction mixture turned red, it was cooled to room temperature. After that, the volume was adjusted to 20 mL with ultrapure water (Milli-Q) to replace any evaporation loss. The yield of reactions using different reducing/capping agents, defined as the fraction of soluble gold that was converted to metal nanoparticles, was determined from measurements of the solution concentrations by inductively coupled plasma optical emission spectrometry (ICP-OES). For this, a calibration curve in the concentration range of 0.5 ppm to 150 ppm was first determined. The samples prepared for concentration determination were read on the equipment at the working concentration (as synthesized, 100 ppm) so that there were no routine losses due to excessive dilutions. The operating conditions of the ICP-OES instrument (Agilent, model 5110, Santa Clara, CA, USA) are listed in Table S1 in Supplementary Information.

### 2.2. Analysis of the Size Distribution and Zeta Potential of the AuNPs

The size distribution of the nanoparticles was determined by dynamic light scattering (DLS—Zetasizer Nano ZS, Malvern Instruments Limited, Malvern, Worcestershire, UK). Measurements were performed in triplicate. Zeta potential was measured six times in a disposable cuvette using the Malvern Zetasizer Nano ZS (Malvern Panalytical Inc., Marvern, UK) size and shape of the nanoparticles were determined using transmission electron microscopy (TEM—JEOL JEM 2100, JEOL USA Inc., Peabody, MA, USA). The samples were prepared by dripping onto a formvar/carbon supported copper grid, mesh 300. The analysis of the nanoparticle size distribution in TEM images was performed according to the protocol published by Zhang and Wang (2023) [22] using ImageJ 1.54 g (64 bit) software (Wayne Rasband and contributors, National Institute of Health, Bethesda, MD, USA). The histograms of size distribution were constructed using Microsoft Excel software (Microsoft Corporation, Redmond, WA, USA).

### 2.3. Conjugation of AuNPs and RBD Protein

The recombinant RBD protein from SARS-CoV-2 was first expressed in mammalian cells and purified as described recently [20]. For the conjugation of RBD proteins on the surface of AuNPs, 10 μg of AuNP suspensions (at 0.52 μ·.μL^−1^) and 10 μg of RBD (at 0.52 μ·.μL^−1^) were added to phosphate-buffered saline (PBS), pH 7.4, in a final volume of 1500 μL using 2.0 mL tubes. The formulations were briefly vortexed for homogenization and then kept at room temperature for 1 h before use for in vivo assays. In vitro adsorption assays were also carried out to evaluate the complexation of AuNPs with RBD protein. The adsorption studies were carried out using 1.5 mL test tubes. In each tube, 20 μL of AuNPs were combined with RBD protein, and PBS buffer (pH 7.4) was finally added to a final volume of 250 μL. The final RBD concentration in each tube was 0.260 μ·.μL^−1^. The test tubes were then vortexed for a few seconds and kept at room temperature. At different adsorption times (every 5 min), one tube was then centrifuged at 10,000× *g* for 10 min to sediment the AuNP–RBD complexes. The supernatants containing the non-adsorbed RBD protein were finally analyzed by UV spectrophotometry (280 nm) using a DS-11 Spectrophotometer (DeNovix Inc., Wilmington, DE, USA) previously calibrated with RBD protein at different concentrations. The experiments were conducted until 1 h of adsorption.

### 2.4. Mice Immunization

Mice were immunized at the isogenic mouse facility of the Microbiology Department, University of Saão Paulo, Brazil. The animals were handled according to the procedures approved by the Committee for the Ethical Use of Laboratory Animals at the Institute of Biomedical Sciences, University of Saão Paulo (protocol number CEUA 6762231118). Six- to eight-week-old female C57BL/6 mice were maintained under pathogen-free conditions. A total of three immunizations were carried out via the intramuscular (i.m.) route in both legs, with an interval of 14 days between doses. Serum samples were collected by submandibular bleeding one day prior to each dose and also on day 48 to investigate the antibody levels induced.

### 2.5. IgG Antibody Levels, Subclass, and Affinity

IgG antibody levels were measured as described by Dalibera et al. (2023) [20]. Briefly, total IgG antibody levels were determined by an indirect ELISA assay using 96-well plates. The plates were sensitized with 200 ng/well of RBD protein for 2 h at 37 °C, washed 3 times using 1X PBS and 0.05% Tween 20 (PBS-T), and finally blocked overnight at 4 °C using 300 µL of PBS -T blocking diluent + 0.5% BSA. As a primary antibody, sera from individual mice were added to the wells, in duplicate, and 2-fold serially diluted starting with 1:50 (Dose 1 and 2) and 1:200 (Dose 3) in 100 μL final volume. The secondary antibody conjugated to peroxidase was added for detection of total IgG anti-HRP 1:10,000 (SIGMA-A2304, St. Louis, MO, USA) and subclasses IgG1 and IgG2c with anti-IgG1-HRP 1:8000 (SIGMA-A9044-1, St. Louis, MO, USA) and anti-IgG2c-HRP 1:4000 (SOUTHERN BIOTECH-1079-05, Birmingham, AL, USA), respectively. To develop the assay, OPD/H_2_O_2_ was used, and the reaction was stopped with 1M H_2_SO_4_. Absorbance was measured at 492 nm on an EPOCH plate reader (BIOTEK, Winooski, VT, USA). All evaluations of antibody affinity to antigen followed a similar ELISA protocol: incubation was performed for 15 min using concentrations of ammonium thiocyanate ranging from 0 to 4 M before incubation with the secondary antibody. In all cases, the titers obtained for the saline group were used to calculate the average result, which was deduced from the titers of all other experimental groups.

### 2.6. SARS-CoV-2-Neutralizing Effects of Anti-RBD Antibodies

The tests conducted followed the protocol described by Pardi and collaborators [23], using Vero cells (ATCC # CCL-81, Manassas, VA, USA) plated at 5 × 10^4^ Vero cells/mL in 96-well plates 24 h before the experiment. The plates containing the cells were incubated at 37 °C and 5% CO_2_ in DMEM supplemented with 10% FBS, 100 μg/mL streptomycin, and 100 U/mL penicillin. This test, known as the virus neutralization test (VNT), was based on the cytopathic effect (CPE) using SARS-CoV-2/human/BRA/SP02cc/2020 (GenBank Nº MT350282.1) [12]. Sera from each immunization group were pooled and heat-inactivated and serially diluted using dilutions from 1:20 to 1:10,240. The diluted serum samples were then mixed 1:1 with 10^3^ TCID50/mL of SARS-CoV-2 diluted in DMEM, and the mixture was pre-incubated at 37 °C for 1 h (for neutralization). The serum and virus mixtures were transferred to a monolayer of confluent cells and incubated for 72 h, after which the plaques were analyzed by light microscopy and evaluated for the presence of cytopathic effects. The reported neutralizing antibody titers were assumed to be the value of the highest serum dilution capable of completely neutralizing viral growth. Additional confirmation was performed by fixing the plates and staining them with black starch (0.1% [*w*/*w*] black starch solution with 5.4% acetic acid and 0.7% sodium acetate) for 30 min. In order to monitor quality, an internal positive control serum was used for each assay. The experiments involving SARS-CoV-2 were all conducted in a Biosafety Level 3 laboratory (BLS3) located at the Federal University of São Paulo, Brazil, following the recommendations of the World Health Organization [24].

### 2.7. Statistical Analysis

The statistical significance was determined using Prism 9 software (GraphPad Software, San Diego, CA, USA). The evaluation included a two-way analysis of variance, followed by a *t*-test with Bonferroni adjustment. All values were reported as individual values or as mean ± SD. Finally, *p*-values below 0.05 were assumed to be statistically significant.

## 3. Results and Discussion

**Figure 1:**
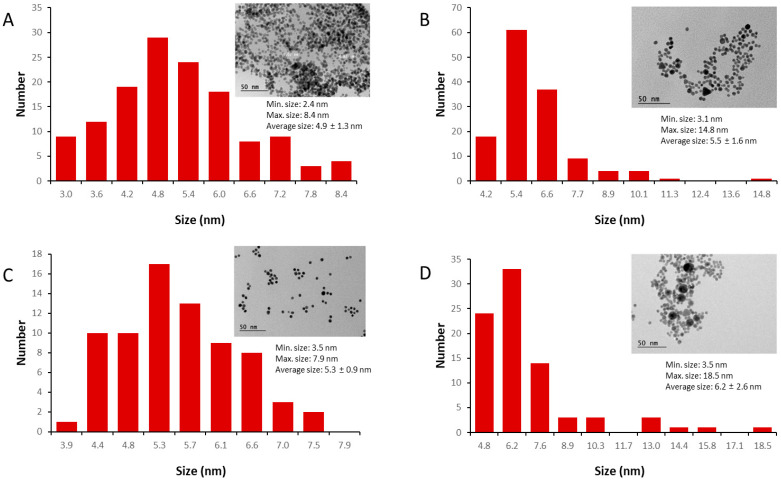
Histograms of size distribution of the different AuNPs obtained by the analysis of the transmission electron microscopy (TEM) images (inserted in all graphics). The AuNPs were synthesized using the following reducing agents: (**A**) citrate (*n* = 135); (**B**) L-cysteine (*n* = 135); (**C**) cysteamine (*n* = 73); and (**D**) L-arginine (*n* = 83); “*n*” is the number of particles analyzed in each image. The scale bar is 50 nm for all images, and the minimum, maximum, average, and standard deviation of the nanoparticles in the samples are also presented.

In this work, we developed a novel method for AuNP synthesis based on a modification of the widely used Turkevich method [21] to produce AuNPs using citrate. The so-called citrate reduction method tends to produce spherical AuNPs with sizes in the range of 20–60 nm [20]. Based on the literature and on our experience, the reproducibility of synthesis methods using conventional stirred reactors (typically flasks) is usually a challenge, mainly due to batch-to-batch variation in the efficiency of reagent mixing (local concentration gradient) and also local temperature, pH, or reactant concentration gradient [25]. Here, the synthesis was performed by the inverse addition of the reducing reagent to HAuCl_4_, associated with the use of ultrasound. Both the inverse addition of reducing agent to the reaction mixture and the use of ultrasound proved to have a synergistic effect on the shape and size of the AuNPs produced, delivering narrower size distribution nanoparticles (Table 1, Figure 1 and Table S1, Supplementary Information). Inverse addition minimizes concentration gradients, while ultrasound generates transient high-energy microenvironments that promote rapid and uniform nucleation, reducing the likelihood of uncontrolled aggregation [18,26,27]. Furthermore, we also exchanged the reducing and capping agents for amino compounds (L-cysteine, cysteamine, and L-arginine), demonstrating high-yield conversions of AuNPs using these agents. In general, the synthesis of the AuNPs using ultrasound was responsible for producing smaller particle sizes than those usually produced using the standard Turkevich method [18,21,26]. However, the synthesis strategy presented here proved to be highly reproducible with respect to the physicochemical properties of the AuNPs. The reaction yields (gold conversion) of the different syntheses were all above 80% (Table 1).

In all cases, the AuNPs presented some dispersion (PDI values between 0.13 and 0.55) and average diameters of less than 13 nm (measured by DLS), and were mostly spherical in shape. The AuNPs produced in the presence of cysteamine presented a positive net surface charge, probably as a result of a free protonated amino group after the Au-S reaction with gold. On the other hand, particles produced using citrate and other amino acids were negatively charged. Cysteamine is a stable aminothiol derived from coenzyme A degradation in mammalian cells, and unlike L-cysteine and L-arginine, cysteamine has no carboxylic acid. Cysteamine bitartrate has been used as a drug to treat cystinosis and cystinuria [28].

The nanoparticle diameters obtained by the analysis of the transmission electron microscopy (TEM) images were similar to the values obtained by DLS, although some differences were seen, especially in the case of L-arginine (Figure 1D). In general, nanoparticle sizes measured by DLS are overestimated compared to TEM ones, since DLS is affected by the strong scattering caused by the presence of larger nanoparticles in the sample. TEM images revealed the presence of large triangular and spherical nanoparticles in the sample synthesized using L-cysteine and L-arginine, respectively. Wider size distributions, with maximum particle diameters within the range of 8.4 to 18.5 nm, were found when using these two amino acids during synthesis, although this was more evident in the case of L-arginine.

Maruyama and colleagues (2015) studied the synthesis of AuNPs using 11 different amino acids [17]. In this case, aqueous solutions of gold (III) chloride acid (1 mL, 1.0 mM) and aqueous solutions of different amino acids (1 mL, 100 mM) were mixed and incubated at 80 °C for 20 min, followed by pH adjustment to 7.1–7.8. Although only the use of L-histidine was studied in detail, only a few of the amino acids used resulted in stable colloidal solutions. L-arginine and L-cysteine were not able to form AuNPs. More recently, Figatt and colleagues (2023) reported the results of systematic studies on the synthesis of AuNPs via a modified Turkevich method using 20 proteinogenic amino acids and 1 non-proteinogenic amino acid (hydroxyproline) as reducing and capping agents [15]. Reactions were performed at boiling temperature under magnetic stirring. Final amino acid and gold salt concentrations were approximately 5 times and 2 times lower than the ones used in the work presented here. A wide range of nanoparticle diameters was found, from 8.5 to 65 nm, depending on the amino acid used. However, in contrast to the results observed in our work, the methodology developed by Figatt and colleagues (2023) did not result in stable gold nanoparticles when using L-cysteine and L-arginine as reducing and capping agents. In the case of L-cysteine, the reaction resulted in the formation of a yellowish precipitate, probably elemental sulfur, the product of the decomposition of cystine formed from the redox reaction of L-cysteine.

**Figure 2:**
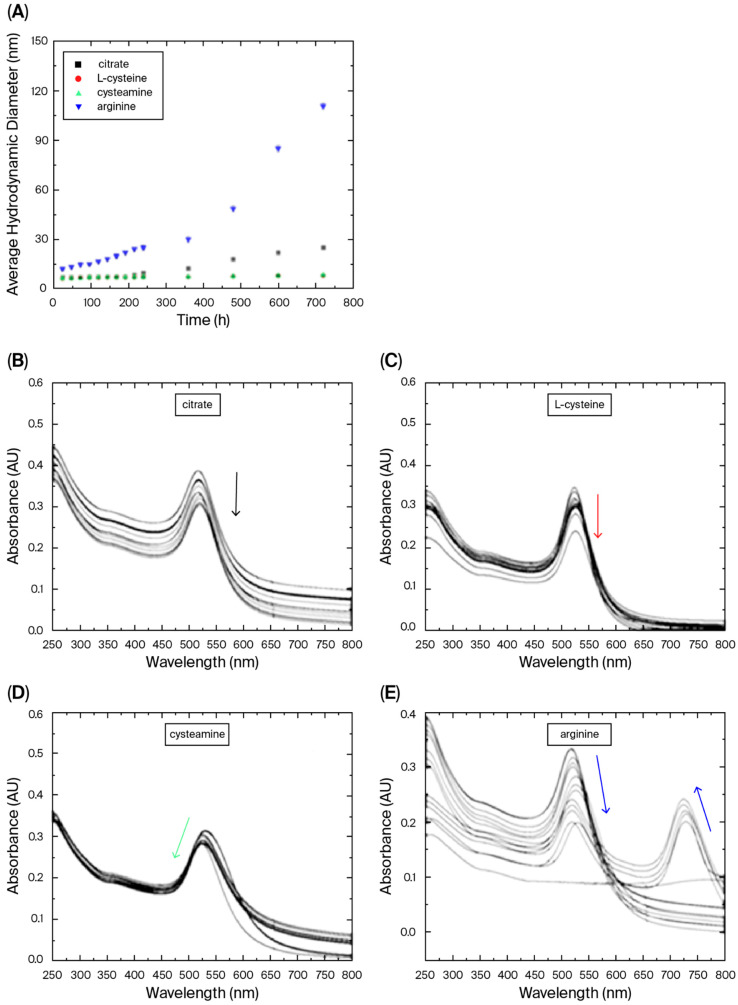
Evaluation of the stability of the AuNPs in terms of the increase in nanoparticle size measured by DLS (**A**). Note that L-cysteine (red dots) and cysteamine (green dots) data presented very similar behavior, with almost identical diameter values during the period of analysis. Absorption spectra of AuNPs synthesized/capped using sodium citrate (**B**), L-cysteine (**C**), cysteamine (**D**), and L-arginine (**E**), at 5 °C, protected from light, after 1 day (upper lines) through 30 days. Colored arrows indicate changes in peak amplitude (absorbance) during the period of study.

In our case, all reducing agents studied were able to generate nanoparticles, and the results indicated that the AuNPs were mostly stable when incubated at 5 °C for 30 days (protected from light), showing small changes in the absorption spectra in the first 190 h (Figure 2). Although the mechanisms of synthesis were not elucidated, we postulate that the success of the synthesis of AuNPs using L-arginine and L-cysteine was increased by the use of ultrasound. Ultrasound creates acoustic cavitation that generates localized “hot spots” with high temperatures and pressures, accelerating chemical reactions and leading to faster nanoparticle formation, and also promoting a more uniform nucleation and growth of nanoparticles [18]. In terms of stability, the DLS analysis indicated an increasing trend of the spectrum over time for AuNPs synthesized using L-arginine. Although the mentioned trend may be a result of the substantial scattering caused by a small fraction of large aggregates in the DLS analysis, the solution color changing from reddish to purple indicates the loss of stability and aggregation compared to the other reducing/capping agents. The AuNPs reduced/capped using L-cysteine and cysteamine were the most stable nanoparticles. As discussed before, cysteamine-coated nanoparticles were the only ones presenting positive charges, probably as a result of the presence of the amino group and absence of the carboxyl group. These changes can have an impact on the immune response generated after the incorporation of AuNPs with antigens.

The main goal of the biological characterization of the AuNPs was to access the ability of the synthesized AuNPs to efficiently bind and deliver the recombinant SARS-CoV-2 RBD antigen to mice, leading to adjuvant effects that may be evaluated either by the increased production of serum (IgG) antibodies or the enhanced ability to neutralize virus infectivity, a characteristic that demands preserved conformation of the target antigen following coupling with the gold particles. The recombinant RBD protein was produced in HEK293 eukaryotic cells, since glycosylation is an important factor for the efficient functional properties of RBD [20,29,30]. The adsorption of the RBD antigen onto AuNPs coated with citrate, L-cysteine, cysteamine, or L-arginine was then evaluated. Our previous work showed that RBD protein interacts strongly with AuNPs [20]. Here, the RBD was associated with AuNPs predominantly by adsorption rather than by deliberate covalent conjugation, since no cross-linking reagents were employed. The binding is mediated by a combination of interactions, including strong thiol–gold coordination (of a covalent nature), together with electrostatic, hydrogen bonding, and hydrophobic forces, as commonly described for protein–AuNP systems. The kinetics of adsorption indicates that binding equilibrium is achieved after 45 min of binding between AuNPs and RBD (Table S2, Supplementary Information). The results also revealed different adsorption capacities (Table 2), defined as the mass of RDB adsorbed per mass of AuNPs in suspension. It is important to note that AuNP–RBD complexes were formed under all tested conditions. The results indicated that the adsorption capacity follows the order AuNP-cysteamine > AuNP-citrate > AuNP-cysteine > AuNPs-arginine. Although the adsorption mechanisms were not studied in this work, the formation of a first layer was not discarded due to the binding of the RBD antigen with the gold surface via thiol groups of cysteine residues, followed by a multilayer formation due to weak reversible interactions [20,31,32,33]. This is especially important in the case of AuNP-cysteamine interaction with the RBD. Adsorption studies have shown that positively charged cysteamine-coated AuNPs exhibit the highest adsorption capacity for the RBD. This is explained by the electrostatic interaction between the positively charged AuNP surface and the negatively charged net RBD protein at neutral pH [30]. Furthermore, this interaction appeared to stabilize the AuNP–protein complexes, as reflected in their higher colloidal stability compared to L-arginine-coated particles. The results also indicated that the smaller adsorption capacity of AuNP-arginine may, at least in part, be related to the larger diameter presented by these particles (Table 1 and Figure 1). Generally, assuming similar shapes, as the particles become larger, their surface area relative to their volume decreases.

According to our calculations, for a 35 kDa RBD protein, there are approximately 1.72 × 10^13^ RBD protein molecules in one microgram. In the same way, in each microgram of 6.5 nm or 12.4 nm gold nanoparticles, there are approximately 3.62 × 10^12^ and 5.19 × 10^11^ nanoparticles, respectively. By dividing these values, we can calculate the numbers of 5 and 33 RBD protein molecules per gold nanoparticle (6.5 and 12.4 nm, respectively) in a formulation using a mass ratio of 1 to 1 RBD/AuNP (*w*/*w*). This calculation assumes that the nanoparticles are perfectly spherical. However, in order to estimate the maximum binding capacities of the different AuNPs, increasing RBD/AuNP ratios were assayed, and the results are presented in Table 2. Assuming the values presented in Table 2, we could estimate the number of 162 and 135 RBD protein molecules per AuNP-cysteamine (6.5 nm) and AuNP-arginine (12.4 nm) gold nanoparticles, respectively, at their maximum loading. This result indicates the existence of a multilayer adsorption phenomenon. These results are consistent with previous results found in the literature, which indicated that at neutral and alkaline adsorption pH values, saturation of the gold surface is not observed and the amount of adsorbed protein exceeds the monolayer film, leading to multilayer adsorption [32]. Sotnikov and colleagues (2019) found adsorption values of 138 BSA (bovine serum albumin, 66 kDa) molecules per AuNP (20 nm in size) during experiments carried out at pH 7.0, and 185 BSA molecules for adsorptions performed at pH 8.0. Under neutral and alkaline pH conditions, a protein corona is formed around the AuNP via interprotein interactions that likely stabilize successive adsorption layers. Wang and collaborators (2014) also studied the adsorption of proteins in gold nanoparticles. The authors proposed a three-step adsorption mechanism, with a first step of reversible adsorption, followed by a second step that includes protein structural modifications and, finally, a third step, when a more stable bind of the protein to the gold surface is formed [33].

**Figure 3:**
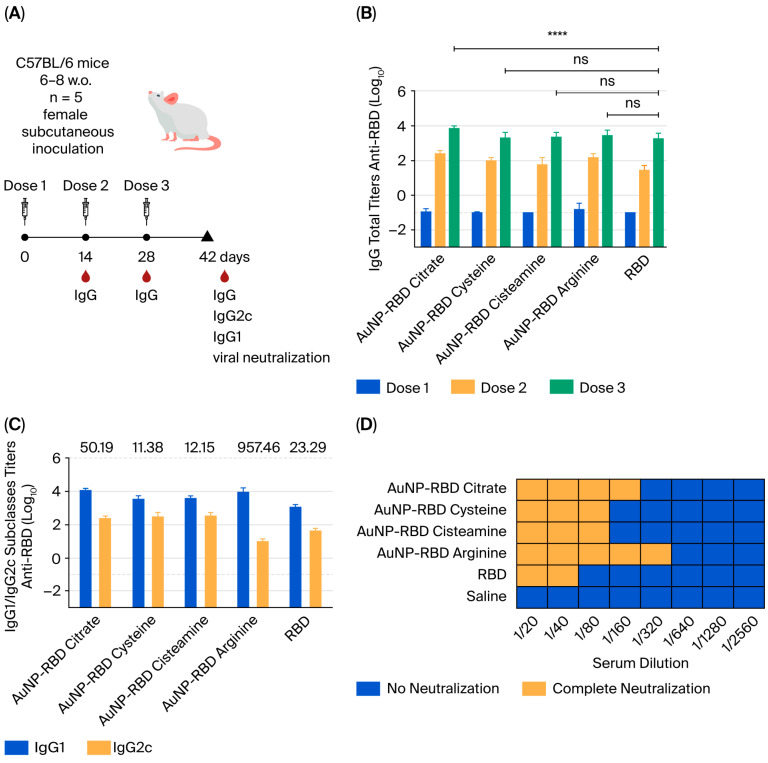
Induction of RBD-specific antibody responses in mice immunized with different AuNP–RBD formulations. The C57/Bl-6 mice (*n* = 5 per group) were administered subcutaneously with 10 µg of RBD alone or conjugated to 10 µg of AuNPs obtained under different conditions. (**A**) Schematic representation of the vaccination regimen. Anti-RBD antibody responses were evaluated in mouse sera by ELISA, measuring total IgG as well as IgG1 and IgG2c subclasses. Sera were collected on days 13, 27, and 42 of vaccine regimen. (**B**) Titers of anti-RBD total IgG after vaccine doses. (**C**) Titers of IgG1 and IgG2c subclasses, assessed 14 days after the third dose (day 42). For both ELISA assays, the values represent the mean ± SE of the absorbance values obtained in an assay with individual murine sera in duplicate for each formulation. The antibody titers measured in the saline control group (mean + 2× SD) were subtracted from those of the other experimental groups. The test cut-off was 0.1, equivalent to 2× MEAN of the blank. Legend of the statistical differences indicated in the graph: **** *p* < 0.0001. The statistical analysis was performed by two-way ANOVA with Bonferroni’s post-test. (**D**) Evaluation of the SARS-CoV-2 neutralization capacity of induced anti-RBD antibodies determined under in vitro conditions. Viral neutralization was performed in duplicate for each dilution, with pools of sera of each group being serially diluted 2-fold. The assay consideres that complete viral neutralization (100%) signifies no cell death. Titers below 1:20 are considered non-neutralizing.

In the next step, we measured the adjuvant properties of the AuNPs following coupling to the recombinant SARS-CoV-2 RBD antigen. Mice were immunized with 3 doses containing 10 ug of AuNPs complexed with 10 ug of RBD (1 to 1 mass ratio), administered 14 days apart (Figure 3A). The results indicated that all tested formulations, including naked RBD, induced antigen-specific antibody responses following parenteral administration to mice (Figure 3B). All formulations benefit from booster doses that increase the induced antibody responses with peak serum RBD-specific IgG titers achieved after 3 doses. The analysis of the IgG subclasses revealed that the IgG1/IgG2c ratio varies in response to AuNPs functionalized with different amino compounds, with varying titers of both subclasses (Figure 3C). AuNP-citrate and AuNP-arginine presented higher IgG1 titers, while naked RBD showed lower response. The highest levels in the IgG1 response are characteristic of an immunomodulatory profile associated with Th2 activation, the main marker of humoral antibody responses [34,35]. This suggests that the AuNP–RBD nanovaccines induced a pattern of predominant humoral immune response.

Next, we assessed the in vitro viral neutralization activity of the sera collected from mice submitted to immunization with different AuNP–RBD formulations (Figure 3D). Neutralizing antibodies are the main correlate of protection for SARS-CoV-2 vaccines, and RBD represents the main target for antibodies capable of blocking binding to cell receptors [18]. The assay considered the presence or absence of virus infection following incubation of live SARS-CoV-2 particles with different dilutions of vaccinated mice sera, and titers below 1/20 were considered non-neutralizing. Our results show that naked RBD has a limited capacity to induce neutralizing antibodies since sera from animals vaccinated with RBD can only block the infection up to a dilution of 1/40. The AuNP–RBD formulations, on the other hand, had an improved capacity to induce neutralizing antibodies, which was remarkably higher in the case of AuNP–RBD arginine (Figure 3D).

An intriguing outcome of this study was that AuNP-arginine, despite exhibiting lower colloidal stability and reduced adsorption capacity, elicited the highest virus neutralization titers. This apparent paradox can be explained by qualitative rather than quantitative effects. The specific interaction of arginine-coated surfaces with the RBD may help preserve key conformational epitopes that are essential for neutralization, even when fewer antigen molecules are bound per nanoparticle. The relatively larger size of AuNP-arginine (12.4 nm) may contribute to a more favorable spatial arrangement and epitope presentation, thereby enhancing B-cell recognition. These results are consistent with previous observations that the quality of antibody responses is more dependent on epitope exposure and preservation than on antigen density alone [20]. Indeed, previous evidence based on a different model antigen showed that the size of the AuNPs is particularly relevant for the induction of antigen-specific antibodies under experimental conditions [36]. It is worth mentioning that similar enhancement of virus neutralization activity of antibodies raised in mice vaccinated with recombinant RBD has been observed under different conditions without the use of AuNPs [37,38,39,40,41]. Furthermore, differences in the size and shape of the AuNPs are known to have a significant impact during in vitro and in vivo studies. Gold nanorods have been reported to be taken up by macrophages in vitro to a lesser extent compared to gold nanospheres [36]. Besides, the kinetic and biodistribution profile of capped gold nanoparticles intravenously injected in the rats may be significantly different depending on the capping strategy used, as presented by Moraes and colleagues [42]. It was shown that peptide capping can significantly increase the hepatic uptake of AuNPs. The size of the nanoparticle may also affect the biodistribution profile of AuNPs in vivo. In the case of AuNPs between 1.4 nm and 5 nm, the accumulation in the liver increases sharply with decreasing size [43]. More recently, Salazar and collaborators (2024) described gold nanoparticle virus-like particles functionalized with full-length SARS-CoV-2 spike protein, highlighting their physicochemical properties and immunogenicity [7]. The results demonstrated that S-AuNP-VLPs consistently enhanced antigen-specific antibody responses compared to the S protein alone. This enhancement included stronger T-cell responses, higher binding antibody titers, and a higher neutralizing capacity of antibodies [7]. Taken together, these findings reinforce the notion that nanoparticle surface chemistry, shape, and size critically impact the immunological outcome beyond simple metrics of antigen loading [44]. These and other aspects regarding the impact of epitope specificity of antibodies raised in mice immunized with AuNPs represent future targets to be tackled.

The in vivo results obtained here demonstrated that amino compound-synthesized AuNPs are able to induce robust antibody responses, and in the case of AuNP-arginine, particularly strong neutralization activity. These findings highlight the potential of this platform for vaccine development and raise the question of its applicability beyond proof of concept. Although this work was performed at the laboratory scale, perspectives for scale-up are promising. Ultrasound-assisted nanoparticle synthesis has already been demonstrated at pilot scale, supporting feasibility beyond bench conditions [45,46]. In general, scaling up can be approached by maintaining consistent specific power input (acoustic power). All other sonochemical synthesis conditions of nanoparticles should also remain unchanged upon increasing the reactor volume. These aspects suggest that rational scale-up strategies can be pursued in future studies to ensure reproducibility for preclinical and industrial applications.

## 4. Conclusions

In this work, we proposed novel gold-based nanovaccine formulations against SARS-CoV-2, consisting of gold nanoparticles (AuNPs) synthesized with amino compounds and combined with the receptor-binding domain (RBD) of the viral spike protein. Using ultrasound in a single-step process, we obtained AuNPs through a reproducible, low-cost, and high-yield methodology in which amino acids or cysteamine acted simultaneously as reducing and stabilizing agents. The resulting AuNP–RBD complexes induced strong antigen-specific IgG responses in mice, with no detectable toxicity, and significantly improved neutralizing activity compared to RBD alone. Interestingly, AuNP-arginine, despite its lower colloidal stability and adsorption capacity, promoted the most effective neutralizing response, underscoring that antigen conformation and epitope presentation are more critical than antigen density for functional immunity. These findings highlight the relevance of nanoparticle surface chemistry and size in shaping immune outcomes and support the potential of ultrasound-assisted amino acid-based synthesis as a practical platform for future nanovaccine development.

## Figures and Tables

**Table 1:** Particle size (measured by TEM analysis and DLS), polydispersity index (PDI), zeta potential, maximum absorbance wavelength, and reaction yield of the synthesis.

Reducing Agent	Size (nm) TEM	Size (nm) DLS ^a^	PDI	Zeta Potential (mV)	λ_máx_ (nm)	Reaction Yield (%)
AuNP-Citrate	4.9 ± 1.3	7.2 ± 0.5	0.198 ± 0.018	−40.1 ± 1.8	525	99.8
AuNP-Cysteamine	5.3 ± 0.9	6.4 ± 1.4	0.138 ± 0.028	+36.5 ± 1.9	525	101.3
AuNP-Cysteine	5.5 ± 1.6	6.3 ± 1.8	0.541 ± 0.128	−25.6 ± 1.3	525	91.2
AuNP-Arginine	6.2 ± 2.6	12.4 ± 2.8	0.313 ± 0.067	−40.5 ± 1.5	530	80.2

^a^ DLS quantification was presented by intensity distribution.

**Table 2:** Adsorption capacity of different AuNPs.

Gold Nanoparticle	AdsorptionCapacity (μgRBD/μgAuNP) *	STD **
AuNP-Citrate	23.7	0.76
AuNP-Cysteine	16.7	0.60
AuNP-Cysteamine	32.5	0.11
AuNP-Arginine	4.13	0.64

* Average value calculated from samples taken at 50, 55, and 60 min of adsorption, as described in Section 2. ** Standard deviation of the three adsorption values described above.

## Data Availability

The authors state that all data underlying the findings are fully available without restriction. All relevant data are within the paper.

## Supplementary Materials

The following supporting information can be downloaded at https://www.mdpi.com/article/10.3390/pharmaceutics17091211/s1, Table S1: ICP-OES instrument operating parameters. Table S2: Adsorption kinetics of RBD protein on the different AuNPs. Figure S1: Transmission electron microscopy (TEM) of AuNPs synthesized at 80 °C and pH 7.0 using as reducing agent: (A) citrate; (B) L-cysteine; (C) cysteamine and (D) arginine. The scale bar is 50 nm for all images.
