# Supplementary material for: Amino Compound-Synthesized Gold Nanoparticles for SARS-CoV-2 Antigen Delivery
Source: Pharmaceutics. 2025 Sep 17;17(9):1211. doi: 10.3390/pharmaceutics17091211 (PMC12473889; doi:10.3390/pharmaceutics17091211)
Supplement: Supplementary file 1 [file pharmaceutics-17-01211-s001.zip › pharmaceutics-3845945-supplementary.pdf]

Supplementary Information

Table S1. ICP-OES instrument operating parameters.

Parameter	Operating condition
Nebulizer	Cross flow
Atomization chamber	Glass-Scott
Power (W)	1,400
Refrigerant gas (L min^{-1})	13.5
Auxiliary gas (L min^{-1})	1.2
Nebulizing gas (L min^{-1})	0.8
Mode	Axial
Sample capture (L min^{-1})	1.0
Analyte wavelengths (nm)	Au 267.595
Measurement time (s)	24

Table S2. Adsorption kinetics of RBD protein on the different AuNPs.

	Nanoparticle			
**Adsorption Time (min)**	AuNP-Citrate C_{RBD} (ug/uL)	AuNP-Cysteine C_{RBD} (ug/uL)	AuNP-Cysteamine C_{RBD} (ug/uL)	AuNP-Arginine C_{RBD} (ug/uL)
0	0.256	0.264	0.261	0.258
10	0.251	0.237	0.191	0.132
15	0.245	0.217	0.158	0.107
20	0.234	0.211	0.132	0.083
25	0.225	0.201	0.099	0.072
30	0.220	0.191	0.081	0.088
35	0.211	0.158	0.053	0.160
40	0.192	0.124	0.022	0.189
45	0.090	0.135	0.008	0.217
50	0.082	0.124	0.008	0.228
55	0.070	0.130	0.007	0.233
60	0.076	0.133	0.006	0.223

* Adsorption studies were performed in 1.0 mL test tubes, in a volume of 250 uL, and at a final AuNP concentration of ~7.8 ng/uL.

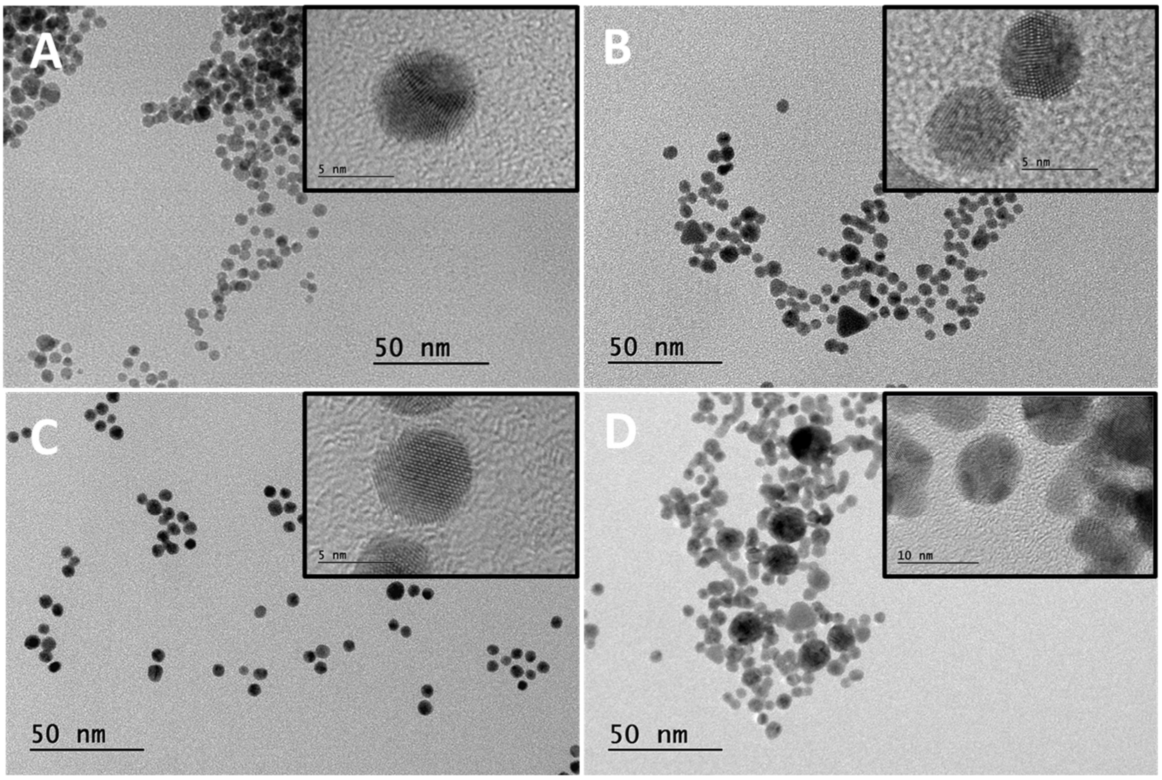

Figure S1. Transmission electron microscopy (TEM) of AuNPs synthesized at 80 °C and pH 7.0 using as reducing agent: A) citrate; B) L-cysteine; C) cysteamine and D) arginine. The scale bar is 50 nm for all images.
